# Analysis of pregnancy outcomes in patients with recurrent implantation failure complicated with chronic endometritis

**DOI:** 10.3389/fcell.2023.1088586

**Published:** 2023-02-13

**Authors:** Jie Li, Xueyao Li, Jinli Ding, Jing Zhao, Jiao Chen, Feng Guan, Haiyu Deng, Mengqi Zhou, Yiling Han, Zhuoni Xiao, Jing Yang

**Affiliations:** ^1^ Center for Reproductive Medicine, Renmin Hospital of Wuhan University, Wuhan, China; ^2^ Department of Pathology, Renmin Hospital of Wuhan University, Wuhan, China

**Keywords:** chronic endometritis (CE), recurrent implantation failure (RIF), *in vitro* fertilizalion-embryo transfer(IVF-EF), antibiotic therapy, platelet-rich plasma

## Abstract

Chronic endometritis (CE) has been considered to reduce uterine receptivity and negatively affect reproductive outcomes for *in vitro* fertilization-embryo transfer (IVF-ET) patients, especially for people undergoing recurrent implantation failure (RIF). To investigate the effects of antibiotic and platelet-rich plasma (PRP) therapy on pregnancy outcomes following frozen-thawed embryo transfer (FET) in RIF patients with CE, endometrial specimens of 327 patients with RIF obtained by endometrial scratching during mid-luteal phase were immunostained by multiple myeloma oncogene-1 (Mum-1)/syndecan-1 (CD138). RIF patients with CE were given antibiotics and PRP treatment. According to the Mum-1^+^/CD138^+^ plasmacytes after treatment, patients were divided into persistent weak positive CE (+) group, CE (−) group and non-CE group. FET was performed and the basic characteristics and pregnancy outcomes of patients in three groups were compared. Among 327 RIF patients, 117 patients were complicated with CE, the prevalence was 35.78%. The prevalence of strong positive was 27.22% and that of weak positive was 8.56%. 70.94% patients with CE converted to negative after treatment. There was no significant difference in the basic characteristics, including age, BMI, AMH, AFC, infertility years, infertility types, number of previous transplant cycles, endometrial thickness on transplantation day and number of transplanted embryos (*p* > 0.05); The positive rate of hCG, clinical pregnancy rate and implantation rate in CE (−) group were significantly increased compared with the weak CE (+) group and non-CE group (*p* < .01), and the live birth rate also improved (*p* < .05). Whereas, the rate of early abortion in CE (−) group was 12.70%, which was higher than that in weak CE (+) group and non-CE group (*p* < .05). After multivariate analysis, number of previous failed cycles and CE remained independent factors associated with live birth rate, while only CE remained to be the independent factor of clinical pregnancy rate. It is recommended to perform CE-related examination for patients with RIF. Antibiotic and PRP treatment can significantly improve pregnancy outcomes of patients with CE negative conversion in FET cycle.

## Introduction

Although a lot of efforts has being spent on improving the procedure of *in vitro* fertilization-embryo transfer (IVF-ET), about 15%–20% of patients also were suffered recurrent implantation failure (RIF), which cause great psychological and economic burdens on patients. The successful implantation depends on various factors, including oocyte and embryo quality, implanted embryo number, endometrial receptivity, and maternal-fetal interface immune factors. How to improve the implantation rate of patients with RIF becomes a hot topic issue which needs to be solved urgently.

Last years, chronic endometritis (CE) has been found in patients with RIF. CE is a persistent chronic inflammatory disorder of the endometrium, characterized by abnormal infiltration of endometrial stromal plasma cells. Patients are usually asymptomatic or oligosymptomatic with mild pelvic discomfort, increased leucorrhea or dysmenorrhea. Although patients with CE has no obvious clinical symptoms, it seriously affects female reproductive health. Studies have reported that the incidence of CE in infertile patients ranges from 2.8% to 56.8% (Kasius et al., 2011; Cicinelli et al., 2018) and those in RIF patients was from 14% to 30.3% (McQueen et al., 2014; Bouet et al., 2016), which suggested that CE is closely associated with pregnancy outcomes.

CE is mainly induced by microbial infection, due to doxycycline has a wide antibacterial spectrum gainst *mycoplasma*, gram-positive cocci and negative bacilli, metronidazole mainly targets anaerobic bacteria, the combination of doxycycline and metronidazole has been considered as the first-line drug in the treatment of CE. Besides, platelet-rich plasma (PRP) contains platelet, platelet derived growth factor (PDGF), transforming growth factor beta (TGF-β), vascular endothelial growth factor (VEGF), epidermal growth factor (EGF) and other cytokines, in view of its regulatory actions on inflammation and local immune, PRP would be an effective alternative approach for patient with persistent CE.

In this study, we focused on discuss the results of antibiotic and platelet-rich plasma (PRP) treatment on RIF women with CE, and then the pregnancy outcomes of freeze-thaw embryo transfer (FET) of those women were analyzed. This research analyzed the effects of antibiotic and PRP therapy on pregnancy outcomes following FET in RIF patients with CE.

## Materials and methods

### Patients

This retrospective study selected RIF patients who received IVF/ICSI-ET at the Reproductive Center of Renmin Hospital of Wuhan University (Wuhan, China) from January 2018 to January 2020. The inclusion criteria were: 1) aged above 20 years and below 40 years old, experienced at least 2 cycles of fresh or freeze-thaw embryo transfer (a total of ≥ 4 high-quality embryos), but had not achieved pregnancy; 2) normal karyotypes of couples; 3) normal uterine morphology and endometrial thickness were showed by color Doppler ultrasound examination, without endocrine disease and thrombophilia. Exclusion criteria were 1) uterine abnormalities; 2) suffering from autoimmune diseases; 3) BMI >28 kg/m^2^ or < 18 kg/m^2^; 4) patients who were previously received antibiotic treatment. This study was approved by the Ethic Committee of Renmin Hospital of Wuhan University and all patients signed informed consent form.

### Endometrial specimen collection

For patients with regular menstruation, vaginal ultrasound was performed 7–9 days after the detection of urinary LH peaks. For patients with irregular menstruation, Femoston (Duphaston, Abbott Biologicals B.V Netherlands, op, qd) were given after was given orally on the first day of menstruation. Vaginal ultrasound was performed on the 21st day of menstruation to examine the endometrium. After the endometrium was determined to be secretory by ultrasound, the endometrial tissue was collected using a disposable endometrial sampler (Jiading Cheng Medical instrument, Jiangsu, China).

All samples were rinsed with hydrochloric acid buffer (PBS), incubated overnight with 10% neutral formaldehyde solution at room temperature and then embedded in paraffin wax.

### Immunohistochemistry staining

The paraffifin-embedded endometrial specimens were cut into sections, and deparaffifinized in xylene and dehydrated through a series of alcohol. Routine HE staining was performed to determine that the endometrium was in the secretory phase, then, immunostaining of Mum-1^+^/CD138^+^ was performed following the manufacturer’s protocol (Jiayuan Tech, Wuhan, Chia).

### Diagnosis of CE

For immunostaining of Mum-1^+^/CD138+were performed by Autostainer Link 48. The positive of CE was defined as the presence of 5 or more plasma cells with Mum-1^+^/CD138^+^ in one of 30 randomly selected high-power fields (HPF; magnigication ×400) (Zargar et al., 2020). Strong positive CE was defined as more than 3 HPFs with ≥ 5 Mum-1^+^/CD138 ^+^plasma cells, while 1–2 HPFs with ≥ 5 Mum-1^+^/CD138+plasma cells was considered as weak positive CE. Less than 5 Mum-1^+^/CD138+plasma cells per HPF in all 30 randomly selected HPF indicated the absence of CE, which was considered non-CE.

### Treatment

Patients with CE were given doxycycline hydrochloride (Jiangsu Lianhuan Pharmaceutical Co., 100 mg, op, Bid, 14 days) and metronidazole (Gold Day Pharmaceutical co., 500 mg, op, Tid, 14 days). To observe the effect of treatment, the endometrium was taken again in next mid-luteal stage for immunohistochemical staining of Mum-1^+^/CD138 ^+^. If the results showed that patients were consistently strong positive, they would be treated with antibiotics and PRP treatment. And the endometrium was taken again.

The PRP was prepared from autologous blood by a modified method (Li et al., 2021). Peripheral bloods were collected from the patients. After centrifugation, the serum, platelets and red blood cells were divided. The serum and platelets were transferred to another tube and centrifuged again. The supernatant was discarded, and the remaining was PRP. Autologous PRP was infused in the uterine cavity with an intrauterine insemination catheter on the 9–10 days of the menstrual cycle and the process was repeated twice times every 3 days.

### Groups

**FIGURE 1:**
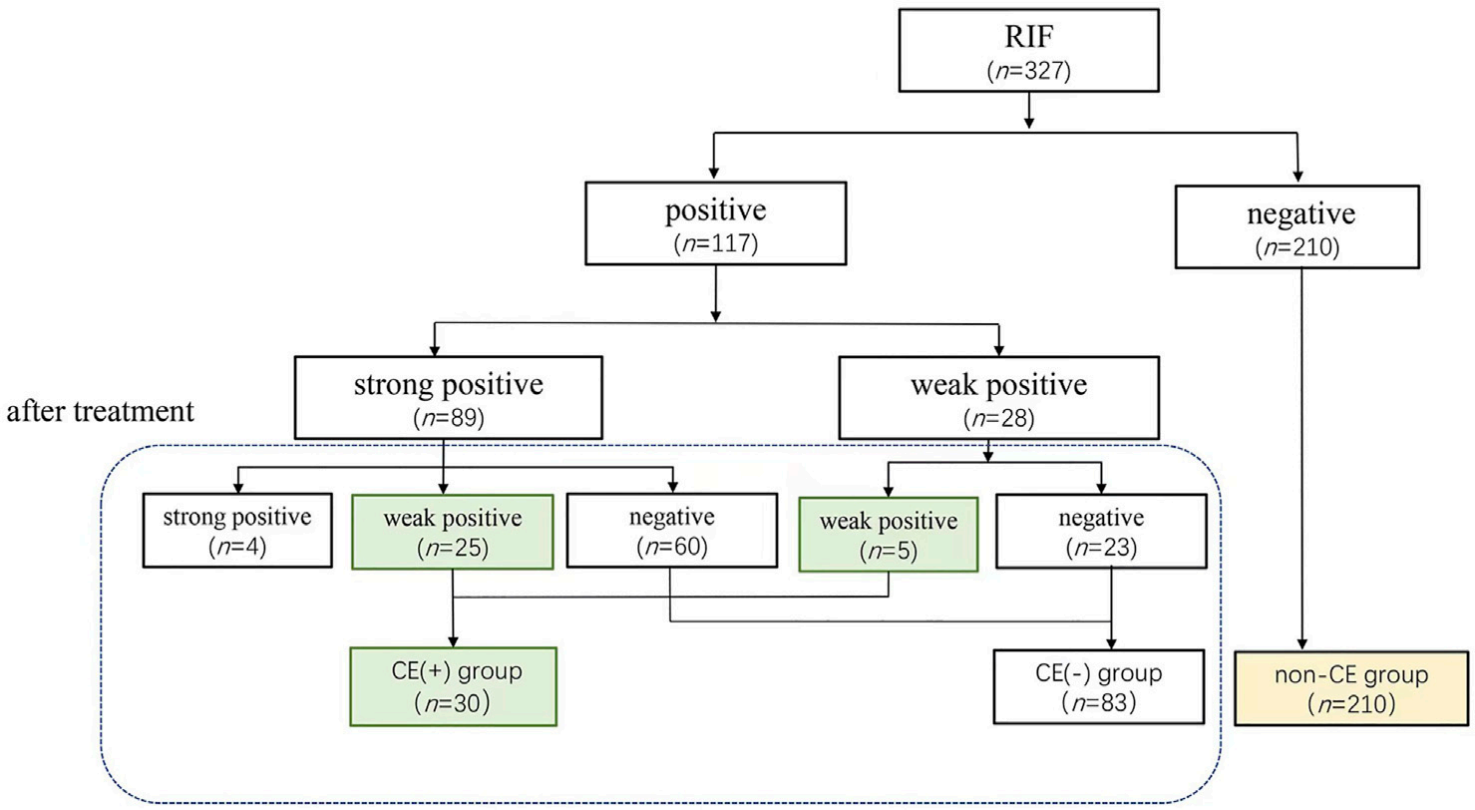
The clinical treatment procedure of RIF/CE patients.

As in the Figure 1, RIF patients with CE received treatment, according to the treatment outcome by antibiotic and PRP, patients with strong and weak positive CE were classified as CE (+) group, patients with non-CE were classified as CE (−) group. RIF Patients without CE were non-CE grup.

### Endometrial preparation

Patients received FET cycle were treated with GnRH-a and hormone replacement therapy (HRT) before embryos transfer. Leuprorelin Acetate Microspheres for Injection (Enantone, Takeda, Japan) was intramuscularly injected on the day 2 of menstruation cycle with a dose of 3.75 mg. Estradiol valerate (Bugale, Bayer, Germany) were administered with a started dose of 4–6 mg/d after 28 days. When the endometrial thickness reached 7 mm, intramuscular injection of progesterone oil (Xianju Pharmaceutical, Zhejiang, China) 40 mg/d was provided for luteal support, after 4 or 6 days, the cleavage-stage embryos or blastocysts were transfrred.

### Embryo transfer and luteal support

Embryos were vitrified and thawed by using the conventional method. Blastocysts with a score of 3BB or better were considered to be high quality (Alpha Scientists in Reproductive and Embryology, 2011). High quality blastocysts were transferred under the guidance of ultrasound. No more than two cleavage-stage embryos or single blastocyst were transferred at one time. Estradiol valerate, intramuscular injection of progesterone oil 40 mg/d and dydrogesterone tablets (Duphaston, Abbott Biologicals B.V Netherlands, 10 mg, op, bid) were given after embryos transfer.

### Pregnancy diagnosis

Serum hCG was measured 10th day or 12th day after embryos transfer, serum hCG level ≥10 IU was considered hCG positive. Clinical pregnancy was defined as the presence of gestational sac on the 30th day after transfer, which was confirmed by trans-vaginal ultrasound. Ectopic pregnancy was defined as with an extra uterine gestational sac. Pregnancy loss before 12 weeks was defined as first trimester abortion, while pregnancy loss during 12–28 weeks was defined as late aortion.

### Outcomes

The main outcomes of this study were the pregnancy outcomes of FET cycle, which included hCG positive rate, clinical pregnancy rate, implantation rate, ectopic pregnancy rate, abortion rate and live birth rate. The hCG positive rate, clinical pregnancy rate and live birth rate were respectively defined as the percentage of hCG positive, clinical pregnancy and live birth relative to the total number of transfer cycles. The implantation rate was defined as the percentage of embryos implanted successfully relative to the total number of embryos transferred. The ectopic pregnancy rate, first trimester abortion rate and late abortion rate were respectively calculated as the number of ectopic pregnancy, first trimester abortion and late abortion divided by the total number of clinical pregnanc cycles.

### Statistical analysis

All date statistical analyses were performed with SPSS 22.0. The continuous measurement data in this article were not following the normal distribution after inspection and were represented by median (interquartile range) [M (P25, P75)]. Comparison between three groups was performed by Kruskal–Wallis H test. The numeration data were expressed in percentage (%), and proportions were compared by chi-square test. Logistic regression analysis was performed to determine the variables that could be independently associated with clinical pregnancy rate and live birth rate. Multivariable logistic regression was performed on variables that were significant at the univariable analysis. Odds ratios (OR) and their 95% confidence intervals (CI) were calculated from the regression model. Statistical significance for all tests was based on a *p*-value <.05.

## Results

In this research, 327 patients with RIF were observed between January 2018 and January 2020.117 patients were confirmed with CE by immunohistochemical staining of Mum-1^+^/CD138^+^. The prevalence of CE was 35.78% (117/327). Strong positive of CE was found in 89 patients (27.22%, 89/327) and 28 patients were weak positive (8.56%, 28/327). After treating with antibiotics, the endometrium of 89 strong positive patients with was taken again at next luteal stage for immunohistochemically detection of Mum-1^+^/CD138^+^. The results showed that 7 patients were consistently strong positive, and 3 of them became weak positive after antibiotics and PRP. Finally, of the 89 CE positive patients, 60 patients (67.42%, 60/89) became CE negative after treatment, 25 (28.09%, 25/89) became CE persistently weak positive, and 4 (4.49%, 4/89) remained CE persistently strong positive after treatment. Among 28 RIF patients combined weak positive of CE, 23 patients (82.14%, 23/28) converted to CE negative after antibiotic treatment and 5 were persistently weak positive (17.86%, 5/28). 83 patients with CE (83/117, 70.94%) converted to negative after treatment.

Except for 4 patients with persistently strong positive of CE, 323 patients received FET again. The process of grouping was shown in Figure 1.

**FIGURE 2:**
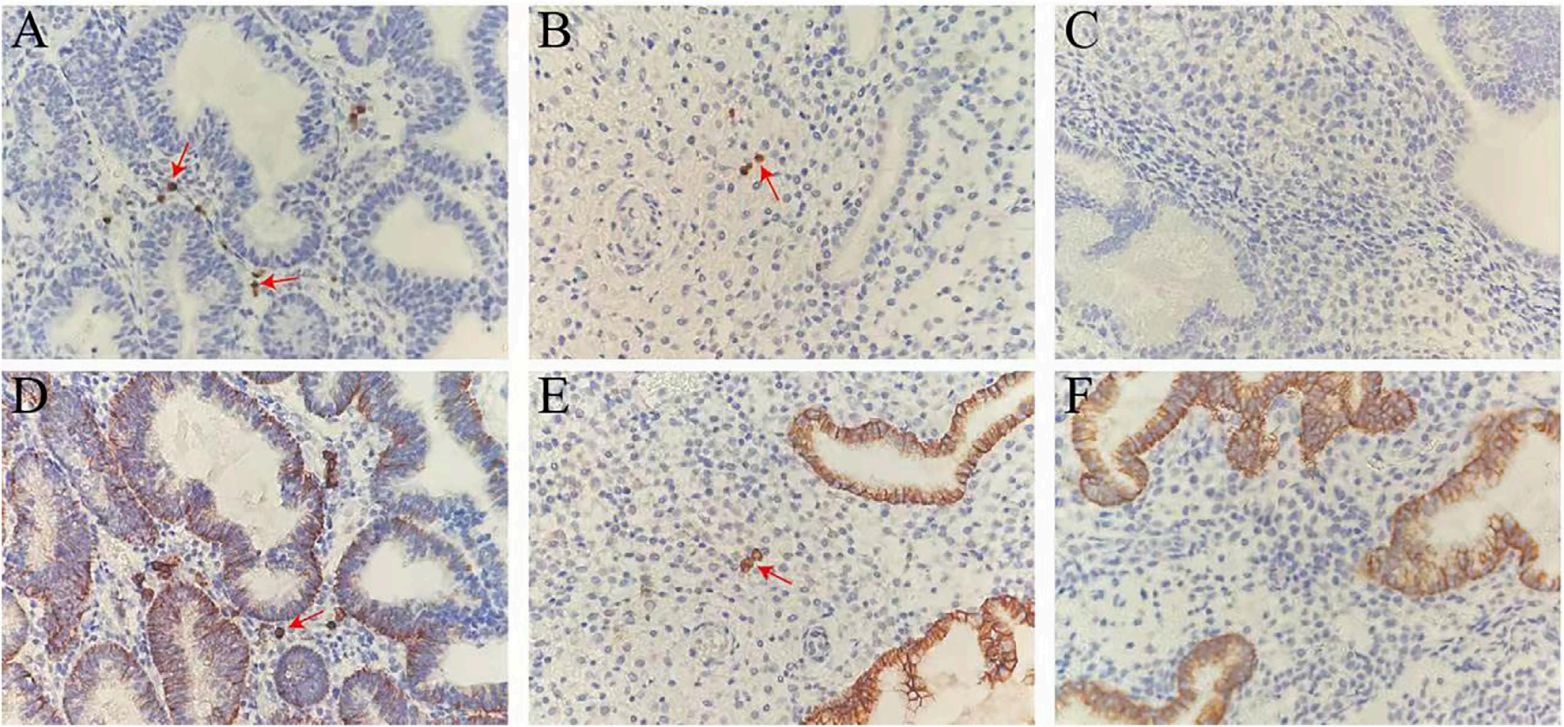
Mum-1^+^/CD138^+^ immunohistochemical staining in the endometrium. **(A–C)** Mum-1^+^ immunohistochemical staining in the strong positive, weak positive and negative groups. **(D–F)** CD138^+^ immunohistochemical staining in the strong positive, weak positive and negative groups. The plasma cells in stroma were positively stained with anti-Mum-1/CD138 (red arrows). Magnification ×400.

As illustrated in Figure 2 ***,*** Mum-1^+^/CD138^+^ plasma cells were expressed in endometrial stroma. The plasma nucleus was round and eccentric, and the condensed chromatin within the nucleus was organized as a wheel pattern. Figures 2A–C respectively represented the Mum-1^+^ immunohistochemical staining in the strong positive, weak positive and negative groups. In these figures, the plasma cell membrane exhibited a strong immunopositivity, and the cytoplasm exhibited weak positive immunostaining with anti-Mum-1. Figures 2D–F were the CD138^+^ immunohistochemical staining in the three groups respectively. Cells were considered to be CD138^+^ plasma cells when the nucleus exhibited a strong immunopositivity with anti-CD138. In addition to plasma cells in endometrial stroma, endometrial epithelial cells also express CD138^+^ on their cytomembrane.

### Baseline clinical characteristics

The baseline clinical characteristics of patients were presented in Table 1. Analysis results showed that no significant difference in age, BMI, AFC AMH, infertility years, infertility types and number of previous transplant cycles was found among three groups (all >.05).

**TABLE 1:** The baseline characteristics of three groups.

	CE (+) group (n = 30)	CE (−) group (n = 83)	Non-CE group (n = 210)	*p*-Value
Age [years, M (P25, P75)]	30.5 (28,33)	32 (29,35)	32 (29.75,35)	>.05
BMI [kg/m2, M (P25, P75)]	21.5 (20.3,22.82)	21.4 (19.8,22.9)	21.4 (20.18,22.8)	>.05
AFC [n, M (P25, P75)]	46.67%	85.54%	70.00%	>.05
	15 (12, 19.25)	15 (13, 17)	15 (13,18)	
AMH [(mIU/mL), M (P25, P75)]	2.89 (1.98,3.8)	2.67 (1.74,3.81)	2.83 (2.10,3.81)	>.05
Infertility time [year, M (P25, P75)]	2 (1,5)	3 (2,4)	3 (2,4)	>.05
Infertility type [n (%)] primary infertility	16 (53.33%)	45 (54.27%)	126 (60.0%)	>.05
secondary infertility	14 (46.67%)	38 (45.78%)	84 (40.0%)	>.05
Number of previous failed cycles [n, M (P25, P75)]	2 (2,3)	2 (2,2)	2 (2,2)	>.05

### Outcomes of FET

In Table 2 ***,*** the analysis results showed that there were no statistically differences in the number of transferred embryos and endometrial thickness on the day of ET among the three groups (*p* > .05). For the pregnancy outcomes, the positive rate of hCG, clinical pregnancy rate and implantation rate in the CE (−) group were significantly increased (*p* < .01) and the live birth rate were also increased (*p* < .05) in CE (−) group. However, the rate of early abortion was 12.70% in CE (−) group, which was higher than that in CE (+) group and non-CE group (*p* < .05).

**TABLE 2:** The outcomes of three groups with FET.

	CE (+) group (n = 30)	CE (−) group (n = 83)	Non-CE group (n = 210)	*p*-Value
Number of embryos transferred [n, M (P25, P75)]	2 (2,2)	2 (2,2)	2 (2,2)	>.05
Endometrial thickness on implantation day [cm, M (P25, P75)]	0.9 (0.78,1.0)	0.9 (0.8,1.0)	0.9 (0.8,1.0)	>.05
hCG positive rate [% (n/n)]	46.67% (14/30)	85.54% (71/83)	70.00% (147/210)	<.01
Clinical pregnancy rate [% (n/n)]	40.00% (12/30)	75.90% (63/83)	61.90% (130/210)	<.01
Implantation rate [% (n/n)]	25.93% (14/54)	51.01% (76/149)	44.44% (164/369)	<.01
Ectopic pregnancy rate [% (n/n)]	0	0	2.31% (3/130)	>.05
First trimester abortion rate [% (n/n)]	8.33% (1/12)	12.70% (8/63)	2.31% (3/130)	<.05
Late abortion rate [% (n/n)]	0	3.17% (2/63)	1.54% (2/130)	>.05
Live birth rate [% (n/n)]	36.67% (11/30)	62.65% (52/83)	58.10% (122/210)	<.05

The results of the univariate and multivariate analysis of possible factors affecting the clinical pregnancy rate and live birth rate in patients were respectively presented in Tables 3, 4. Number of previous failed cycles and CE were both associated with a significantly difference in clinical pregnancy rate and live birth rate in univariate analysis. After multivariate analysis, number of previous failed cycles (OR = 0.549; 95% CI: 0.350–0.861; *p* = 0.009) and CE (OR = 0.437; 95% CI: 0.198–0.967; *p* = 0.041) remained independent factors associated with live birth rate, while only CE (OR = 0.373; 95% CI: 0.171–0.812; *p* = 0.013) remained to be the independent factor of clinical pregnancy rate. In addition, age, BMI, AFC, AMH, infertility time, infertility type, number of transferred embryos and endometrial thickness were not significantly associated with the pregnancy outcomes.

**TABLE 3:** Logistic regression analysis of factors affecting the clinical pregnancy rate.

	Univariate analysis			Multivariate analysis		
Variables	*p*-Value	OR	(95%CI)	*p*-Value	OR	(95%CI)
Age	.660	.987	(0.932, 1.046)			
BMI	.097	.927	(0.847, 1.014)			
AFC	.357	.974	(0.920, 1.030)			
AMH	.832	.984	(0.852, 1.138)			
Infertility time	.888	.993	(0.897, 1.098)			
Infertility type	.118	1.448	(0.910, 2.306)			
Number of previous failed cycles	.035	.631	(0.412, 0.968)	.072	.668	(0.431, 1.037)
Number of transferred embryos	.108	1.532	(0.910, 2.578)			
Endometrial thickness	.511	1.580	(0.404, 6.175)			
CE	.007	.345	(0.160, 0.746)	.013	.373	(0.171, 0.812)

**TABLE 4:** Logistic regression analysis of factors affecting the live birth rate.

	Univariate analysis			Multivariate analysis		
Variables	*p*-Value	OR	(95%CI)	*p*-Value	OR	(95%CI)
Age	.661	.988	(0.934, 1.044)			
BMI	.364	.961	(0.881, 1.048)			
AFC	.460	.979	(0.927, 1.035)			
AMH	.728	.975	(0.847, 1.123)			
Infertility time	.855	.991	(0.898, 1.093)			
Infertility type	.350	1.238	(0.791, 1.939)			
Number of previous failed cycles	.004	0.528	(0.340, 0.820)	0.009	0.549	(0.350, 0.861)
Number of transferred embryos	.158	1.447	(0.866, 2.417)			
Endometrial thickness	.850	1.136	(0.303, 4.250)			
CE	.020	.396	(0.182, 0.862)	0.041	0.437	(0.198, 0.967)

## Discussion

RIF has always been a hot issue in the clinical work of assisted reproduction. Therapeutic strategies have been performed to improve the pregnancy outcomes of unexplained RIF, which included individualized ovulation induction regimens, laboratory quality interventions, improvement of transplantation methods, immunomodulatory therapies (Nyborg et al., 2014), granulocyte colony stimulating factor (G-CSF), autologous peripheral blood mononuclear cells (PBMC), lymphocytic active immunotherapy, PRP and endometrial receptivity array (Nyborg et al., 2014; Wurfel, 2015; Farimani et al., 2017; Li et al., 2017; Tan et al., 2018; Sfakianoudis et al., 2019). Despite these developments, some patients still suffer from RIF. The studies reported that CE had been closely related to the pregnancy outcomes of IVE-ET (Bouet et al., 2016; Vitagliano et al., 2018; Zhang et al., 2019), it was reported that CE affected embryo implantation by altering endometrial receptivity. The researchers considered that there were some plasma cells in the endometrial stroma, but the abnormal number of plasma cells would lead to CE, which affected embryo implantation negatively. The mechanism would be related to that microbes infected the uterine cavity and released pathogenic agents, resulting in abnormal expression of chemokines and adhesion factors in endometrial epithelial cells, stromal cells and vascular epithelial cells, ultimately disrupted the endometrial microenvironment, which would reduce the endometrial receptivity and led to the failure of embryo implantation (Kitaya et al., 2016).

CE can be diagnosed by some methods (Cicinelli et al., 2018; Liu et al., 2019; Liu et al., 2020; Xu et al., 2020), including etiological cultivation method, hysteroscopy, pathological HE staining combined with immunohistochemistry and sequencing.

By the pathogenic examination, we can find that the main pathogenic bacteria of CE were *Streptococcus species, Escherichia coli, Enterococcus faecalis*, and followed by *Mycoplasma* (Kitaya et al., 2016). In hysteroscopy, CE presents as endometrial stromal edema, micropolypsis, irregular endometrial hyperplasia, and focal or diffuse endometrial hyperemia. Abnormal plasma cells can be found in the endometrial stroma by pathological examination. However, conventional HE staining, which searches for plasma cells according to morphology, is easy to miss round and spindle plasma cells, and has subjective judgment bias, which affects the diagnosis of CE.

CD138 as one of the specific markers of plasma cells, can effectively compensate the deficiency of morphologic examination and increase the detection rate of plasma cells from 6% to 52% (Kitaya et al., 2016). CD138^+^ is expressed in different stages of normal lymphocytes, such as pre-B cells, immature B cells and plasma cells. It is also expressed in epithelial cells, embryonic interstitial cells, vascular smooth muscle cells and endothelial cells. It is difficult to identify plasma cells in endometrial specimens when both plasma cells and epithelial cells appear brown. Mum-1 is a transcription factor of mature differentiation of B lymphocytes into plasma cells and is expressed primarily as brown granules on the nucleus. Mum-1^+^/CD138^+^ immunohistochemical staining has been recently used in clinic, because of its high sensitivity and easy operation. Double labeling of markers, accurate location, clear background, no specific staining, greatly improved the intensity and accuracy of plasma cell staining, convenient interpretation, effectively make up for the deficiency of morphological examination.

In our research, we used Mum-1^+^/CD138^+^ immunohistochemical double staining method to stain plasma cells. The result showed that the prevalence of strong positive of CE and weak positive of CE and non-CE in RIF patients was 27.22%, 8.56% and 64.22% respectively.

CE is mainly induced by intrauterine microbial infection. Clinical treatment should be given according to the results of bacterial culture or microbial detection in the uterine cavity. Because of the limitations of bacterial culture or microbial detection in uterine fluid and endometrium, patients with RIF with CE were treated with empirical antibiotics. Doxycycline has a wide antibacterial spectrum and is effective against *mycoplasma*, gram-positive cocci and negative bacilli. Multiple studies recommended doxycycline as the effective drug in the treatment of CE, which has been used and achieved good therapeutic efficacy (Johnston-Macananny et al., 2010; McQueen et al., 2014; Kitaya et al., 2017). Metronidazole mainly targets anaerobic bacteria, the combination of doxycycline and metronidazole has been considered as the first-line drug in the treatment of CE. Also, PRP as an effective alternative approach for patient with RIF and persistent chronic endometritis (Sfakianoudis et al., 2019).

In our study, CE was treated by doxycycline combined with metronidazole. For persistent CE, the patient received antibiotics and autologous intrauterine platelet-rich plasma treatment. The result of immunohistochemical staining for Mum-1^+^/CD138^+^ showed that 4 patients continued to be strong positive, 30 patients became weak positive, and 83 patients turned negative after treatment, with a conversion rate of 70.94% (83/117). Compared with the non-CE group, the clinical pregnancy rate and live birth rate of the CE (+) group were decreased, while these two outcomes and the positive rate of hCG were statistically increased in CE (−) group. However, the early abortion rate was higher in the CE (−) group, which suggested that embryo factors still need to be considered for the cause of RIF. Genetic testing is recommended before embryo transfer.

Our analysis showed that antibiotic and PRP had potential to improve pregnancy outcomes in women with CE/RIF. However, given the limited number and variable quality of the included studies, the current evidence should be interpreted with caution, and more randomized clinical trials with larger sample sizes re needed.

## Conclusion

CE is an important factor leading to RIF, it is recommended to perform CE-related examination for patients with RIF. Furthermore, this study demonstrated that the pregnancy outcomes of CE/RIF patients can be improved by antibiotic and PRP treatment. More multi-center studies with a large sample are required to investigate the appropriate microscopic diagnostic criteria for CE, avoiding overtreatment or under-treatment. For patients with persistent positive CE, endometrial bacterial culture or DNA sequencing can be considered to identify the infected microorganisms, which is would be helpful to the accurate use of antibiotics in clinica procedure.

## Data Availability

The original contributions presented in the study are included in the article/Supplementary Material, further inquiries can be directed to the corresponding authors.
